# Investigating hierarchical control among functional networks disrupted by opioid use disorder using effective connectivity

**DOI:** 10.1162/IMAG.a.1373

**Published:** 2026-09-17

**Authors:** Brianna Austin, Danielle L. Kurtin, Katherine Herlinger, Alexandra Hayes, Lexi J. Hand, Leon Fonville, Raymond G. Hill, David J. Nutt, Anne R. Lingford-Hughes, Louise M. Paterson

**Affiliations:** Division of Psychiatry, Faculty of Medicine, Imperial College London, London, United Kingdom; Department of Metabolism, Digestion and Reproduction, Faculty of Medicine, Imperial College London, London, United Kingdom

**Keywords:** effective connectivity, neuroimaging, task-fMRI, clustering, addiction

## Abstract

Opioid use disorder (OUD) poses a significant public health challenge. Developing a better understanding of the brain mechanisms and potential markers of OUD would facilitate the development of therapeutic interventions. While we recently showed that between-network connectivity is disrupted in people with OUD compared with healthy controls, it remains unclear what mechanisms may drive these disruptions and how dysfunctional interactions propagate across large-scale functional networks. To advance the mechanistic understanding of the disrupted processes in OUD, this study used Effective Connectivity (EC) to quantify disrupted hierarchical control among functional networks governing cognition, attention, and reward. We also explored whether whole-brain patterns of EC were effective markers to distinguish people with severe OUD from controls. We hypothesized that the ventromedial network (VMN) would drive dysfunction in cognitive and attentional networks in people with OUD. Task-fMRI data were collected from healthy controls (HC; n = 22) and OUD participants on methadone maintenance treatment (OUD; n = 25), during a heroin cue reactivity (CR) and monetary incentive delay (MID) task. Following brain parcellation (214 regions) and network assignment (7 functional networks), EC was quantified using large-scale nonlinear Granger causality. Dimensionality reduction was performed using uniform manifold approximation and projection, followed by hierarchical density-based spatial clustering of applications with noise to assess whether EC patterns could form clusters corresponding to group labels. Contrary to our hypothesis, the VMN did not drive dysfunction in cognitive and attentional networks. Instead, edges with significantly stronger EC in HC vs. OUD participants were within and between the control, somatomotor, and default mode networks. EC patterns were unable to form distinct clusters that corresponded to clinical groups; instead, clusters separated by task. Little evidence was found that disrupted VMN-mediated reward drove dysregulated whole-brain function in OUD.

## 1 Introduction

Opioid use disorder (OUD) is a chronic relapsing condition characterized by the persistent compulsion to obtain and consume an opioid, accompanied with significant psychological and physical distress when opioid availability is prevented (Blanco & Volkow, 2019). OUD is often characterized by substantial clinical heterogeneity, including high rates of polydrug use and comorbid psychiatric conditions (Santo et al., 2024). These co-occurring features are not incidental but form part of a broader clinical presentation and reflex a complex clinical phenotype. Although harm minimisation initiatives and opioid substitution therapies (OST, e.g. methadone and buprenorphine) have shown some success, relapse rates remain high. For example, over 60% of individuals relapse within 6 months post-treatment (Hayes et al., 2020) and many individuals continue to use illicit opioids ‘on-top’ of their OST. Consequently, there is a need for improved relapse prevention strategies.

One way to develop more effective therapies is to improve the understanding of the neural underpinnings of OUD. Assessing connectivity between brain regions is a valuable approach to evaluate how the brain coordinates resources to support neural processes underlying addictive behaviors. Functional magnetic resonance imaging (fMRI) studies consistently report altered connectivity patterns in individuals with OUD compared with healthy controls (HC) (Liu et al., 2011; Wei et al., 2021). Network-based models of brain function emphasize how disrupted communication across neural circuitry may contribute to relapse risk and abstinence (Liang et al., 2015), as well as cognitive and affective disturbances characteristic of substance use disorders (SUDs) (Hayes et al., 2020; Kurtin et al., 2025; Tolomeo & Yu, 2022; Zilverstand et al., 2018). Key brain networks often reported disrupted in addiction include the following: the ventromedial-prefrontal cortex network (VMN), which supports reward and loss processing; the executive control network (ECN), involved in high-level cognition and coordinating top-down cognitive control; the salience network (SN), which governs prioritization and orchestration of responses to stimuli; and the default mode network (DMN), which supports self-reflection and introspection (Breukelaar et al., 2017; Dunlop et al., 2017; Gratton et al., 2018; Lin et al., 2017; Smallwood et al., 2021).

Balanced interactions among these networks are essential for adaptive decision-making. In SUDs, connectivity between the VMN and the ECN, SN, and DMN is commonly observed to be reduced, while interactions with the DMN show region-specific increases and decreases which may relate to an overvaluation of substance-related rewards and reduced sensitivity to negative consequences (Koob & Volkow, 2016; Kurtin et al., 2026; Tolomeo & Yu, 2022; Zhang & Volkow, 2019). While disrupted functional connectivity (FC) observed in heroin addiction is often observed during resting-state, it is arguably more useful to evaluate differences in brain function during performance of tasks, as these can engage and elicit specific processes associated with addiction and relapse (Chen et al., 2022; Finn, 2021). For example, tasks that probe reward processing, a central component driving the initial consumption of substances and the development of dependence, can reveal the brain correlates of reward, and inform allocation and utilisation of neural resources.

Cue reactivity (CR) tasks are well-validated paradigms that elicit brain activity associated with reward, memory, and attentional processes in response to salient (i.e. drug-related) vs neutral cues (Dieterich & Endrass, 2022; Hill-Bowen et al., 2021; Zilverstand et al., 2018). In response to heroin-related drug cues, people with OUD show greater magnitude of the blood oxygenation level dependent (BOLD) signal in the mesocorticolimbic system, notably nucleus accumbens (NAc), amygdala, anterior cingulate cortex, and thalamus (Huang et al., 2024; Liu et al., 2011; Zilverstand et al., 2018). These regions comprise the VMN, which subserves both reward and anti-reward processes (i.e., aversive negative states like dysphoria, anxiety, and withdrawal related distress) (Ciaramelli et al., 2021; Koob, 2013; Koob & Le Moal, 2008). Hyperactivity in the VMN in OUD during cue reactivity is thought to reflect increased incentive salience of drug-related stimuli.

By contrast, the monetary incentive delay (MID) task is used to explore non-drug reward processes; specifically neural responses governing anticipation and receipt of monetary reward. A meta-analysis found that individuals with SUD, including OUD, exhibited lower BOLD activation in the NAc during anticipation of monetary reward but greater activation in response to receipt of the reward (Luijten et al., 2017). This pattern of hypoactivation followed by hyperactivation was interpreted as evidence of reward deficiency, and supports the learning-deficit theory, which suggests that individuals with SUD may struggle to effectively associate predictive cues with reward value (Nestor & Ersche, 2023; Ngetich et al., 2024). Together, this indicates an imbalance in reward-related neurocircuitry in SUD, with inappropriate allocation of neural resources resulting in disproportionate responsivity to salient vs. non-salient rewarding stimuli. Indeed, disrupted activity among VMN regions during drug- and non-drug-related reward processing is one of the most reliable neural biomarkers of SUD, including OUD (Ekhtiari et al., 2024; Moeller & Paulus, 2018; Sangchooli et al., 2024). It has been suggested that dysregulated reward/anti-reward processing in the VMN may be a key driver of disruptions in broader network connectivity patterns and potentially reinforcing maladaptive behaviors (Dunlop et al., 2017). VMN instability may contribute to relapse vulnerability and is therefore a potential treatment target.

In support of this hypothesis, our previous work used mutual information FC (miFC), a measure that quantifies how much information the activity in one brain region provides about another and captures both linear and nonlinear statistical dependencies (Kurtin et al., 2026; Li, 2022). Using miFC, we found that during both CR and MID tasks, methadone-maintained individuals with OUD generally had weaker miFC compared with controls (Kurtin et al., 2026). More interestingly, during the MID task, stronger miFC was observed from the VMN compared with both the control and DMN in OUD vs. controls, further supporting a key role for the VMN as a hub for neural resource allocation during reward processing (Kurtin et al., 2026).

While these earlier works provided insights into dysfunctional network coordination, FC analyses do not inform the direction of influence. In task based fMRI, estimates of directed interactions are influenced by the temporal structure of the task. For example, experimental paradigms such as the CR and MID tasks involve structured stimulus presentation that can simultaneously engage multiple regions between and within across functional networks. As a result, regions may exhibit temporally ordered dependencies driven by common task related inputs, rather than solely by direct inter- and intra- regional signalling. As a result, effective connectivity (EC) methods applied to fMRI may capture directed statistical relationships in task-evoked BOLD dynamics, reflecting how activity in one regions predicts activity in another within the context of the task. Effective connectivity (EC) is a methodology to estimate directed interactions between brain regions, offering insight into how activity in one region may statistically predict activity in another (Friston, 2011). Large scale nonlinear Granger causality (lsNGC) employs nonlinear state space reconstructions and multivariate analysis to overcome limitations of some other EC methods that are unsuited to the discrete, noncontinuous, and sparse samples evident in fMRI data (Shorten et al., 2021; Wismüller et al., 2021).

The primary aim of this study was to extend our previous observations of disrupted between-network connectivity in OUD by assigning directionality to FC within networks of interest and evaluating whether there is a hierarchy of control among functional networks disrupted in OUD compared with healthy controls (HC). We hypothesised that OUD participants would typically show weaker EC than HC, indicative of a compromised ability to optimally coordinate neural processes during tasks probing responses to drug (heroin cue) and non-drug (monetary) rewards. In line with suggestions by Dunlop et al. (2017) and Kurtin et al. (2025), we hypothesised that the VMN would be the “dominant network of influence”; that is, that significant differences in EC would be driven by regions in the VMN. This influence would be observable either by virtue of the VMN having the highest proportion of significant edges contributing to EC differences, or by many edges with significantly higher EC in OUD vs. HC showing significantly stronger connections from regions in the VMN to regions in other networks. As an exploratory aim we investigated whether an unsupervised clustering model could distinguish people with OUD from HC. If successful, measures of feature importance would inform which networks (and their corresponding processes) provide maximal distinction between groups.

By characterising directed, network level interactions in a whole-brain analysis this work moves beyond traditional region-of-interest analyses to reveal how disrupted hierarchal control may underlie impaired reward processing in OUD. Understanding which networks exert dominant influence could shed insight into the mechanisms driving maladaptive behaviour. These findings could help identify specific neural circuits as potential targets for therapeutic modulation, guiding the development of network-informed interventions for people with OUD.

## 2 Materials and Methods

Data from the Neural Correlates of Reward and Emotion (NCORE) study were used for this study (Fonville et al., 2021). The NCORE study’s primary aim was to investigate the neural correlates of reward and emotional processing in individuals with severe opioid use disorder receiving methadone, compared with matched healthy controls (HC).

The study was approved by the West London & Gene Therapy Advisory Committee (REC:19/LO/0971) and followed the Declaration of Helsinki principles. All participants provided written informed consent. Informed consent was obtained from all participants for being included in the study.

### 2.1 Participants

OUD participants (n = 29) receiving methadone maintenance therapy (up to 60 mg/day) were recruited from community-based drug and alcohol services in London and surrounding areas. HC (n = 22) were recruited through volunteer databases and public advertisements. All participants were >18 years old, and the OUD group had a confirmed severe OUD diagnosis according to the Diagnostic and Statistical Manual of Mental Disorders, 5th Edition (DSM-5), and were receiving a stable dose of methadone (able to maintain the same stable dose throughout the study).

Exclusion criteria included: (1) positive alcohol breath test, pregnancy test, or urine drug screen for drugs of abuse (excluding cannabis due to the extended half-life of cannabinoid metabolites), (2) use of relapse prevention medication, (3) current medical condition that could affect study integrity, (4) current or history of severe enduring psychiatric disorder (including psychotic disorder, bipolar, excluding depression or anxiety), and (5) current alcohol or substance dependence disorder (excluding nicotine). Additional exclusion criteria for the OUD group included regular top up usage of opiates, heroin or other illicit substances, and for the HC group included current DSM-5 psychiatric disorder. As all OUD participants were receiving methadone maintenance therapy, the current study reflects individuals in treatment rather than untreated OUD populations. Full details of inclusion and exclusion criteria can be found in Fonville et al. (2021).

### 2.2 Study protocol

Participants attended the Imperial Clinical Research Facility for a screening visit to determine eligibility, and then up to two experimental study visits in a randomised cross-sectional design, separated by at least 5 days. In each experimental session, participants were given their usual prescribed dose of methadone, then administered either a placebo or 320 mg of aprepitant, approximately 2 hours prior to an fMRI scan at the Imperial Clinical Imaging Facility (Fonville et al., 2021). The data we report in the current paper are only obtained from the placebo session. Participants were familiarized with the fMRI neurocognitive tasks and completed questionnaires before attending the MRI scanner. This current study focused on two of the in-scanner tasks, the CR and MID tasks.

### 2.3 Cue reactivity task (CR)

Participants passively viewed stimuli presented in a block design, with 10 blocks of images in total (5 blocks containing heroin-related and 5 containing neutral cues) according to Fonville et al., 2021). Heroin-related imagery depicted different stages of drug use and related paraphernalia. Images were selected and co-produced with an independent focus group of individuals with lived experience of OUD, to reflect local population heroin usage. Neutral cues were matched for contrast, hue, complexity, brightness, and content (e.g., faces and hands to the heroin-related cues). Each block had between 5 and 7 images which were shown for 5 s each, followed by a jittered interval lasting 200–500 ms. Neutral blocks were followed by heroin blocks, with a 15s fixation cross rest block in between. OUD participants completed 2 task runs, while HC completed 1 task run, with each run lasting 8 min 20s. Different images were used at the second experimental visit, but otherwise the task design was identical. To ensure comparability between groups and to avoid potential confounds related to repeated cue exposure or habituation, only the first run of the task is included in the analyses reported in the current study.

### 2.4 Monetary incentive delay task (MID)

The MID was modified from Knutson et al. (2000) and measures responses to the anticipation and receipt of monetary reward (Knutson et al., 2000; McGonigle et al., 2017). The task begun with an anticipatory cue indicating whether participants could win money, lose money, or neither. Then, participants were instructed to respond as quickly as possible to a target stimulus with a button press. The anticipatory cue indicated one of the three trial types: win, lose, and neutral. In win trials, fast responses earned £0.50, while on loss trials, slow responses resulted in a loss of £0.50. Neutral trials involved neither reward nor penalization. Altogether, there were 18 win, 18 neutral, and 6 loss trials, which were designed to maintain approximately 66%-win trial accuracy with average total winnings of ~£5. The tasks’ event-related design featured long mini-blocks, each spanning several repetition times in length, with a total run time of 7 m 18s. Each trial had three phases: anticipatory cue, target response, and feedback. An anticipatory cue notified participants of the trial type, followed by a blank black screen for 2–4s. After, the target stimulus (star) was shown in the target phase, in which participants responded by pressing the button. The feedback phase immediately followed for 0.5s, where participants were shown outcome feedback in accordance with their response. Total winnings were then displayed for 2s. After feedback, a fixation cross appeared for 2.4–4.4s before the next trial. The target stimulus duration varied for each participant, ranging between 150–300 ms dependent on their trial-by-trial accuracy, and increasing or decreasing by 10 ms to maintain a 66% accuracy rate using a staircase algorithm. In win and neutral trials, the target stimulus duration was initially 280 ms, whereas loss trials were initially 240 ms to ensure participants experienced losses, therefore heightening the trials’ reward salience. Participants underwent training trials before fMRI scanning to ensure task comprehension.

### 2.5 fMRI preprocessing and acquisition

MRI data were acquired over the course of an hour, with a 3T Siemens Magnetom Verio. T1-weighted MPRAGE high-resolution anatomical images were obtained (TR = 2300 ms, TE = 2.98 ms, TI = 900 ms, flip angle = 9°, FOV = 256 mm, voxel size = 1 mm^3^). Four functional sequences were obtained, beginning with a resting state sequence followed by the MID, Evocative Images, and CR tasks. Only the functional data from the MID and Cue Reactivity sequences were analysed in this work. Functional images were acquired with a T2*-weighted gradient echo EPI sequence (TR = 1500 ms, TE = 30 ms, flip angle = 62°, FOV = 192 mm, voxel size = 3 mm^3^, 54 slices,) with in-plane (GRAPPA) acceleration factor of 2 and a multiband acceleration factor of 2.

Functional MRI preprocessing was conducted as described in Kurtin et al. (2026). Preprocessing included using the fMRI Expert Analysis Tool (Version 6.0) from the FMRI of the Brain Software Library (FSL). Boundary-based registration (BBR) of functional data to structural T1w space was performed with 6 degrees of freedom (dof). Interleaved slice-time correction and brain extraction were conducted using FSL’s’ Brain Extraction Tool. FSL’s MCFLIRT performed motion correction with 24 parameters (transformation and rotation in the x, y, and z axis, with their temporal and spatial derivatives). Spatial smoothing was conducted with a 5 mm full width at half maximum Gaussian kernel filter. Framewise Displacement (FD) was computed to assess whether participant’s data met inclusion criteria (<20% volumes with FD>0.9 mm). FSL’s Independent Component Analysis classified components (ICA AROMA) as either signal or motion-related noise, the latter of which were regressed out using the *regfilt* function (Jenkinson et al., 2012; Smith et al., 2004; Woolrich et al., 2009).

### 2.6 Brain parcellation and timeseries extraction

Cortical regions were defined by transforming the 200-parcel Schaefer atlas to subject space using ANTs nonlinear registration by using a symmetric diffeomorphic (SyN) warp. The warp was estimated using the probability mapping metric with a Gaussian regularisation kernel (σ = 2 mm, regularisation weight = 0.5) optimized over 100*300*120 iterations at successive resolution levels. The result warp field and affine transformation were applied to the atlas image using nearest-neighbour interpolation, preserving integer region labels in subject space. In the Schaefer atlas, each parcel label is assigned to one of seven functional networks as defined by Yeo et al. (2011) (Schaefer et al., 2018).

Fourteen subcortical regions were anatomically defined using FreeSurfer’s *recon-all* and assigned to the functional network most strongly supported by literature. Of the seven Yeo resting state networks, the limbic network most closely aligns with the VMN in both the cortical regions it includes (e.g., OFC) and the functions it supports (reward and emotional processing). Accordingly, subcortical regions associated with the VMN—the NAc, thalamus, caudate, putamen, and amygdala—were labeled as part of the limbic network (Dunlop et al., 2017). The hippocampus was added to the DMN given its well-established functional coupling with core DMN nodes during episodic memory and internally directed cognition (Barnett et al., 2021; Ranganath & Ritchey, 2012). The pallidum to the somatomotor network has its role in motor function (Grillner et al., 2005; Neumann et al., 2017). Regional timeseries were defined as the mean signal from all voxels with the region and were extracted using the *fslmeants* function. Each timeseries was z-scored to centre mean 0 and ±1 standard deviation.

### 2.7 Large-scale nonlinear granger causality

EC was quantified using large-scale nonlinear Granger causality (lsNGC), which infers directed statistical dependencies between timeseries (Wismüller et al., 2021). Granger causality relies on the premise that if the prediction quality of one time series improves when using past values of another time series, then the latter is considered to have a causal influence (Granger, 1969). Unlike classical linear GC, lsNGC estimates conditional GC using phase-space reconstructions of multivariate time series, while considering confounding variables and limited observations (Wismüller et al., 2021). Prediction quality is compared using estimation models: one using information from only the suspected influential timeseries and another containing all states except the suspected influential timeseries. If the model including the suspected influential timeseries has a better prediction quality compared to that of the exclusionary model, then suspected influential timeseries is considered to Granger-cause the target time series, indicating a directional and potentially causal relationship exists between the two timeseries. The prediction quality of these models is compared using F-statistics, and a pairwise directed affinity matrix can be derived that quantifies EC between all brain regions across the timeseries (Wismüller et al., 2021).

Full lsNGC implementation and mathematical formulation are available in Wismüller et al. (2021) and the Supplementary Methods (see section 1.1).

### 2.8 Dominant network of influence

In the current study, the dominant network of influence is defined a descriptive characterisation of hierarchical control and was based on two approaches. First, as the network with the greatest degree, as determined by the number of regions within the network showing significantly different EC between HC vs. OUD groups. Pairs of regions with significantly different EC between groups are referred to as edges and were identified using a linear mixed-effects model (see section 2.11 below). Intra- and inter-network edges were counted when computing the degree of each network. Second, we averaged the EC for all edges between each network, and tested whether there was a significant difference in between-network EC. For networks with significantly different EC between networks, the dominant network of influence was identified as the network with the largest effect size.

### 2.9 Uniform manifold approximation and projection

Uniform Manifold Approximation and Projection (UMAP) was used to reduce the dimensionality of the pairwise EC matrices for subsequent clustering analysis. UMAP is a nonlinear dimensionality reduction technique that preserves both global and local structure in high-dimensional data, making it well-suited for exploring patterns in brain connectivity networks. The pairwise connectivity values from flattened affinity matrices were scaled using MinMaxScaler prior to UMAP processing to ensure a normalised input range between 0 and 1.

The UMAP parameters were optimized through hyperparameter tuning (see Supplementary Methods section 1.2. for further details). The optimized target was a combination of trustworthiness score and silhouette score, which are metrics of dimensionality reduction and clustering quality. The trustworthiness score quantifies how well the local neighborhood relationships in the high dimensional data are preserved in the reduced dimensional space, with higher values indicating better retention and representation of local structure (van der Maaten, 2009; Venna & Kaski, 2001). The reduced embeddings were then used as inputs for subsequent clustering analysis.

### 2.10 Hierarchical density-based spatial clustering of applications with noise

Following dimensionality reduction, hierarchical density-based spatial clustering of applications with noise (HDBSCAN) identified clusters within the UMAP-reduced embeddings. HDBSCAN is an unsupervised**,** density-based clustering algorithm that detects clusters of varying densities without a prior specification of the number of clusters (Campello et al., 2013). In HDBSCAN, density is defined by the concentration of data points within a given neighborhood, where clusters are formed in areas with a high density of points. HDBSCAN identifies clusters based on how closely data points are packed together, which allows for structures of varying densities to be identified. For details regarding HDBSCAN’s classification logic (e.g., core, border, noise points) and hyperparameter tuning, see Supplementary Methods section 1.3.

Silhouette score and cluster purity were computed to assess clustering performance. Silhouette scores measure the separation of the identified clusters, and cluster purity quantifies the homogeneity of cluster compositions relative to known group labels (HC vs. OUD) (Herrmann et al., 2024; Rousseeuw, 1987). We evaluated the proportion of noise points and the distribution of group labels within noise and cluster groups.

### 2.11 Statistical analysis

Between-group differences in participant clinical and demographic data were analyzed with the Chi-Squared test for nominal data**,** the Mann-Whitney U test for non-parametric data, and the unpaired t-test for parametric data. Linear mixed effects models (LMEMs) were employed to evaluate whether between network EC connectivity significantly differed between HC vs. OUD participants in each task.

For each participant, mean EC values were calculated for each of the 49 possible within- and between-network pairs. For each network pair, EC values were entered into a table containing three variables: EC, Group (HC vs OUD), and the subject ID. Group and subject were treated as categorical variables. The following model was fit for each network pair:

EC∼Group+(1|Subject)

Where group was treated as a fixed effect and subject was a random intercept. F-statistics and p-values for the Group effect were extracted from each model using ANOVA. Partial eta squared (η²_p_ ) was calculated as a measure of effect size:

$$\eta_{p}^{2}=\frac{F\times DF_{1}}{F\times DF_{1}+DF_{2}}$$

Benjamini–Hochberg false discovery rate (FDR) correction was applied to control for multiple comparisons across the 49 network pairs, with significance defined as FDR-corrected p < 0.05, and effect size captured by Hedge’s G. For networks with significantly different EC between networks, the dominant network of influence was identified as the network with the largest effect size.

To assess the importance of each feature’s contribution to cluster separability, Kruskal-Wallis tests evaluated the differences in feature distributions across multiple clusters, and to identify the features which contributed the most to cluster differentiation. Mann U-Whitney tests compared the distributions of these top-ranked features between clustered data and the noise points.

All statistical analyses were conducted in MATLABR2023b.

## 3 Results

### 3.1 Clinical and demographic information

HC (n = 22) and OUD (n = 29) participants receiving methadone maintenance treatment completed the placebo scanning sessions. Four OUD participants were excluded from analyses as they did not meet the study head motion threshold (>20% of volumes in either task with framewise displacement >0.9 mm). Participants were well matched for age and gender, but not education, smoking status, or current psychiatric diagnosis (Table 1).

**Table 1.** Clinical and demographic characteristics of the OUD and HC participants.

	HC	OUD	Group comparisons p-value
n	22	25	-
Age (years)	46.9 ± 11.0 (30–65)	43.0 ± 9.8 (19–60)	p = 0.270
Females, n (%)	3 (13.6%)	6 (24.0%)	p = 0.400
Years of education	15.8 ± 2.5 (11–20)	13.2 ± 2.6 (9–20)	p < 0.001
Caucasian, n (%)	18 (81.8%)	15 (60.0%)	p = 0.130
Methadone dose (mg/d)	-	31.0 ± 14.4 (3–60)	-
Age of first heroin use	-	25.4 ± 9.2 (11–49)	-
Total years on methadone	-	7.2 ± 7.1 (1–30)	-
DSM-5 Heroin*	-	10.4 ± 0.8 (8–11)	
DSM-5 Crack cocaine* ^	-	7.0 ± 4.7 (0–11)	-
Smoking status, n (%)	18 (81.8%)	24 (96.0%)	p = 0.170
Current Psychiatric Diagnosis, n (%)	0 (0%)	10 (40.0%)	p = 0.002

Data shown as mean ± standard deviation (range). Smoking status includes those who were current or ex-smokers and/or vapers.

* DSM-5; Severity score at time of dependence.

^ NB: 5 participants did not meet criteria for mild, moderate, or severe crack use disorder.

### 3.2 No robust differences in EC and network dominance observed between HC and OUD

#### 3.2.1 CR task

**Fig. 1.**
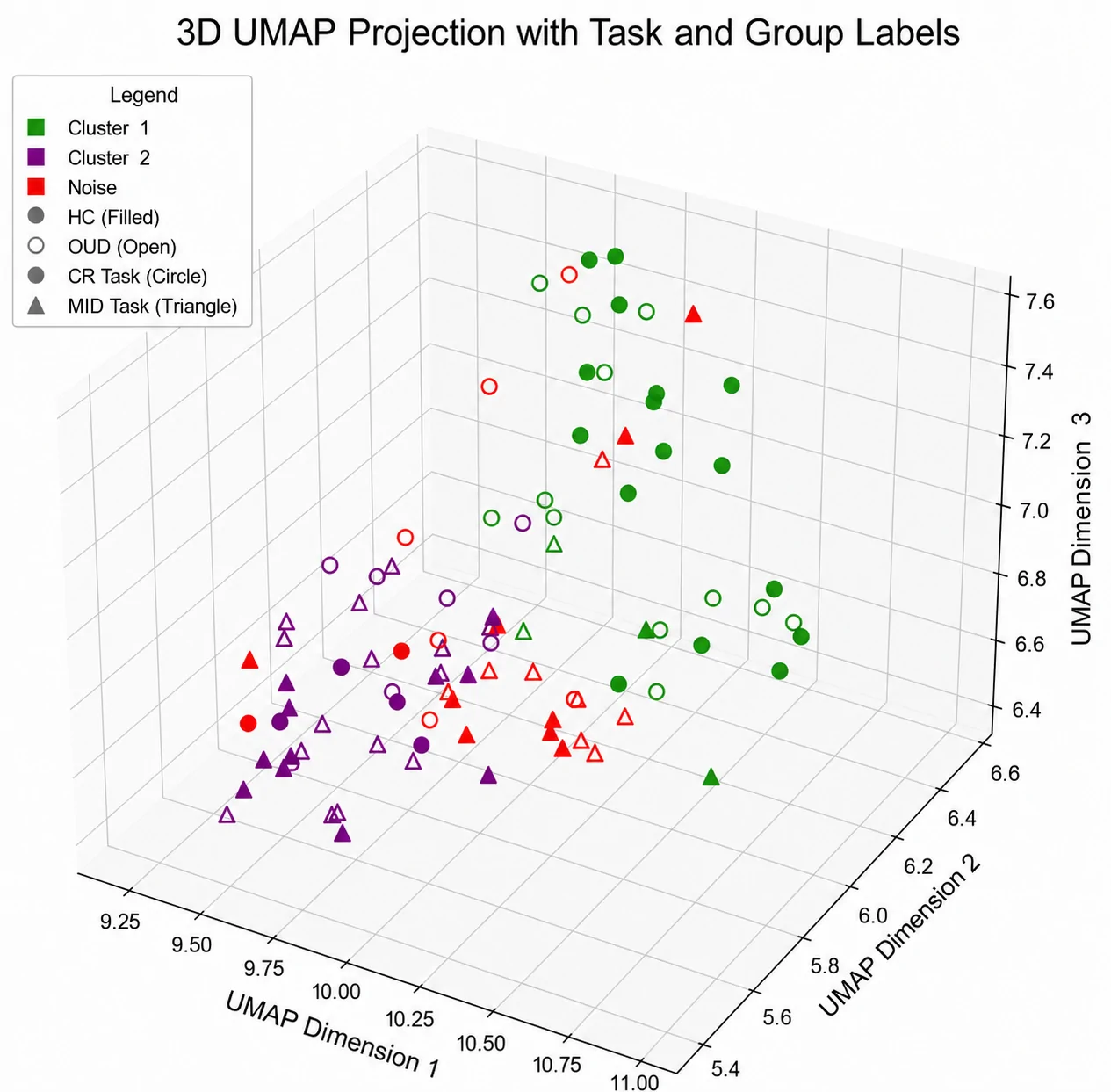
3D UMAP Projection of EC features across 94 observations (one per task from each of the 47 participants), with clusters identified by HDBSCAN. This figure shows the underlying structure within the dataset, showing how samples cluster based on similar EC and the spatial separation of participants. Colors correspond to the identified clusters (Cluster 1: green, Cluster 2: purple, Noise: red). Closed circles represent HC, while open circles represent OUD participants. Closed shapes also correspond to task, with circles representing the CR task, and triangles the MID task.

In the CR task, there were seven between-network EC pairs that showed significant (p < 0.05) pairwise differences in EC between OUD and HC participants at the uncorrected level (Table 2). These involved connections between salience ventral attention, somatomotor, control, limbic, dorsal attention, and visual networks. The largest effect sizes were observed for the salience ventral attention →somatomotor (Hedges’ g = 0.677), control→somatomotor (Hedges’ g = 0.658), and limbic→somatomotor (Hedges g = 0.649) (Table 2, Fig. 1). However, none of these pairwise differences did not survive FDR correction for multiple comparisons.

**Table 2.** The top 7 between-network EC pairs showing the corrected and uncorrected group differences for the CR task.

Network pair	Uncorrected p-value	Hedge’s G	Corrected p-value	Partial eta^2^	F-statistic
Salience ventral attention → somatomotor	0.022	-0.665	0.281	0.111	5.597
Control → somatomotor	0.026	-0.647	0.281	0.105	5.287
Limbic → somatomotor	0.028	-0.638	0.281	0.103	5.154
Control → salience ventral attention	0.030	-0.631	0.281	0.100	5.027
Dorsal attentional → salience ventral attention	0.044	-0.584	0.281	0.088	4.316
Salience ventral attention → salience ventral attention	0.046	-0.577	0.281	0.085	4.206
Control → visual	0.050	-0.570	0.281	0.084	4.101

The full between-network EC pairs group differences for the CR task can be found in the Supplementary Results (see section 2, Table 2).

#### 3.2.2 MID task

In the MID task, there were no significant pairwise differences in EC between OUD and HC participants before and after FDR correction for multiple comparisons.

The full between-network EC pairs group differences for the MID task can be found in the Supplementary Results (see section 2, Table 3).

**Table 3.** The top 7 between-network EC pairs showing the uncorrected group differences for the MID task.

Network pair	Uncorrected p-value	Hedge’s G	Corrected p-value	Partial eta^2^	F-statistic
Somatomotor → somatomotor	0.090	-0.488	0.963	0.063	3.011
Salience ventral attention→ somatomotor	0.117	-0.450	0.963	0.054	2.555
Visual → limbic	0.162	0.400	0.963	0.043	2.021
Dorsal attention → limbic	0.163	0.399	0.963	0.043	2.014
Limbic → limbic	0.182	0.381	0.963	0.039	1.840
Somatomotor → salience ventral attention	0.257	-0.323	0.963	0.029	1.321
Limbic → visual	0.273	0.312	0.963	0.027	1.233

### 3.2 Unsupervised clustering of EC patterns across participants is driven more strongly by task rather than clinical group

To assess whether EC patterns in HC and people with OUD would form clusters driven by clinical group, we first applied dimensionality reduction to reduce the complexity of the high-dimensional data while preserving local neighbourhood structure and then applied clustering to evaluate whether participants with similar patterns formed meaningful groupings.

Following UMAP, the dataset was reduced from 94 (47 participants aggregated over both tasks) by 45,796 dimensions (derived from all unique edges across participants and task aggregates) to 94 by 10 dimensions. The trustworthiness score for the UMAP-reduced space was 72.0, indicating a moderate preservation of local neighbourhood structure in the reduced space. After applying HDBSCAN to the UMAP-reduced data, the silhouette score was 64.2% and cluster purity was 60.0%, suggesting moderately well separated and interpretable groupings.

Two main clusters and a noise cluster were formed by HDBSCAN (Fig. 1). The cluster composition for each cluster is shown in Table 4. Notably, both groups were present in each cluster, indicating that the derived patterns likely reflect the effect of task rather than being specific to either HC or OUD.

**Table 4.** Cluster composition by diagnostic group and task.

Cluster	HC	OUD	CR	MID
1	18 (56.2%)	14 (43.8%)	28 (87.5%)	4 (12.5%)
2	15 (40.5%)	22 (59.5%)	11 (29.7%)	26 (70.3%)
Noise	11 (44.0%)	14 (56.0%)	8 (32.0%)	17 (68.0%)

We show the extent to which each cluster is composed of HC or OUD participants, as well as CR or MID task. The composition of each cluster is described in the number of observations assigned to each cluster, with percentages representing the proportion of that cluster occupied by each diagnostic group or task label. Since each of the 47 participants contributed two EC profiles (one during the MID task and another during the CR task), the clustering and UMAP projections were performed on 94 observations.

#### 3.2.1 Cluster-specific effective connectivity signatures

To further interpret the clusters, we examined the 10 most important discriminative EC features for each cluster based on deviation from the group mean (Supplementary Results, Table 1). The identified connections spanned multiple functional brain networks, suggesting patterns of network-level dysregulation that differ across clusters (Supplementary Fig. S1). Notably, connections including the salience, somatomotor, and default mode were highly represented across clusters, albeit with different cross-network pairings. Cluster 1 showed salience, somatomotor and dorsal attentional inter- and intra-network connectivity, which may reflect a subtype of participants with altered interoceptive/motor integration, possibly linked to habitual behaviour patterns. Cluster 2 showed a mix of attentional, salience, and default mode network and several interhemispheric and cross-network links, suggesting possible dysregulation of task negative/task positive balance. The noise cluster showed connections more heavily involved in visual-sensory and control network involvement, possibly reflecting greater heterogeneity, participants with atypical connectivity patterns or artifacts because of data quality issues (Table 4).

## 4 Discussion

Here, we sought to investigate whether disruptions in between-network EC in OUD vs HC would concentrate in the VMN, providing stronger evidence for the urge-to-action trajectory proposed by Dunlop et al. (2017). Previously, we showed participants with OUD had stronger connectivity during portions of each task thought to engage processes disrupted by OUD (Kurtin et al., 2026). For example, results from the drug cues > neutral cues during the CR task and the win outcome > neutral outcome during the MID task showed OUD participants had stronger connectivity between the VMN and other networks in OUD vs HC participants. These results presented a network connectivity profile in OUD aligning with the strong incentive salience of drug cues compared with HC, and when considered the replication in the MID task, in the relationship between affective processing and task strategy/performance more broadly (Kurtin et al., 2026). However, these results could not directly investigate whether the disrupted connectivity between the VMN to other networks was driving the between-network differences observed in OUD vs HC. One aim of this work was to apply EC and investigate whether we observed evidence to support the “urge-to-action” trajectory proposed by Dunlop et al.

However, we did not find evidence to support our hypothesis that the VMN would drive differences in EC between HC and OUD. Indeed, we did not identify any significant differences in pairwise EC between HC and OUD participants in either task. Given this absence of evidence, not evidence of absence, any interpretation of these results is limited. For example, we might be underpowered to detect such differences at the level of correction required for this number of comparisons, or the effects are smaller or more distributed than our design was sensitive to detect.

Also, in contrast to our hypothesis, distinct clusters did not form based on group label. Instead, our clustering results showed that network coordination for each task shapes functional topology to a greater extent than group differences. Together, these findings may suggest there is a systems-level, within- and between-network disruption in how participants with OUD coordinate the feedforward and feedback communication that supports task-dependent processing.

### 4.1 No robust differences in EC were observed in HC and people with OUD

Our study found group differences in EC between OUD and HC across multiple functional networks in the CR task. However, these differences did not survive correction for multiple comparisons. Findings were consistent with previous reports of weaker connectivity among networks such as DMN, salience, and control networks in individuals with OUD vs. controls. The pattern of weaker between-network connectivity at the uncorrected level may suggest in OUD participants a difficulty coordinating processes governed by the DMN and ECN. For example, Zhang and Volkow (2019) suggested that decreased connectivity within the DMN may indicate individuals with OUD have a heightened interplay between these networks, potentially affecting their ability to make decisions without interference from internally driven processes (Zhang & Volkow, 2019). However, as none of these effects survived correction for multiple comparisons, this may indicate between group differences were distributed and of modest magnitude rather than focal and robust. Interestingly, we observed no significant results before or after correction for multiple comparisons in the MID task. One reason for this difference in nominal significance between the tasks may be due to differences in tasks’ design. The CR task involves prolonged exposure to drug-related cues in a block design, which may allow for more sustained activation of multiple brain networks in people with SUD vs HC over time (Batouli & Sisakhti, 2020; Fonville et al., 2021). For people with OUD, this sustained activation could lead to more dysregulated or unstable patterns of network connectivity, whereas HC may maintain more coordinated connectivity. Consequently, this prolonged engagement may increase sensitivity to subtle group-differences in EC more stable and detectable changes in EC across various brain regions.

Contrastingly, the MID task employed an event-related design with discrete anticipation and outcome phases within each trial that are salient to both groups (Knutson et al., 2001). It is possible that the variability introduced by the event-related design, combined with the salience of the task to both groups, potentially led to fewer detectable significant edges (Batouli & Sisakhti, 2020). Another possibility is that the CR task more directly engages processes disrupted in addiction than the MID task does. It is important to mention that our EC analyses used the entire task time series rather than isolating task relevant epochs (e.g., cue presentation or reward anticipation). This approach allowed us to capture overall connectivity patterns but may conflate state-specific processes with general task engagement.

### 4.2 The reward network is not driving dysfunction in people with OUD

Our hypothesis that the VMN would be the dominant network driving between-group EC differences was inspired by Dunlop et al. (2017), which proposed reliable, robust disruptions in VMN activity and connectivity not only reflect the impaired reward and antireward processing in SUD but also drive disruptions in other functional networks and their corresponding processes. By quantifying EC during tasks that engage the VMN-governed processes, this study evaluated the evidence supporting the framework outlined by Dunlop et al. (2017). However, we did not find strong evidence that the VMN was the primary driver of EC differences between HC and OUD participants in either task, even at the uncorrected level. Instead, in the CR task, nominal between group differences in EC were observed in connections involving salience, somatomotor, and control networks, but these effects did not survive correction for multiple comparisons, so patterns should be interpreted with caution. Interestingly, in the MID task, we observed no between group differences in EC at either the nominal or corrected level. As such, we did not observe a clear pattern of VMN related alternations during monetary reward processing at the level of large-scale network connectivity. Taken together, the findings from this study do not support the characterisation of OUD by VMN-centred network disruption. This result was surprising given the volume of literature describing the critical role of reward/anti-reward and associated VMN network dysfunction in OUD specifically, and SUD more broadly. For example, reviews have suggested that targeted inhibition of the VMN, with repetitive transcranial magnetic stimulation, appears promising in preclinical studies for mitigating the maladaptive incentive salience observed in substance dependence (Dunlop et al., 2017). Moreover, empirical work demonstrated that modulating VMN activity can reduce drug craving in people with OUD (Dunlop et al., 2017; Rezai et al., 2025). However, these findings primarily concern VMN activity and FC, rather than directionality. The present results suggest that VMN engagement alone may be insufficient to explain broader network dysfunction in OUD.

Finally, it is important to acknowledge that all OUD participants were on methadone maintenance therapy (MMT), and methadone may modulate VMN reactivity in OUD. Preliminary findings from this cohort have shown heightened BOLD responses to heroin cues in MMT relative to healthy controls, suggesting that heightened drug-related cue reactivity remained despite MMT (Herlinger et al., 2023). Therefore, the absence of EC differences in OUD participants vs. HC may not be related to the effects of MMT on VMN cue reactivity, or widespread network connectivity. Nevertheless, it is worth considering that the MMT in the OUD participants may have reduced sensitivity to detect VMN directed estimates specifically and should be considered when interpreting the absence of widespread between-group differences and VMN-related findings. Future work in treatment-naïve populations would help clarify whether MMT contributes to the null VMN findings observed in the present study.

### 4.3 Cluster separation is more influenced by task than clinical group label

Our exploratory clustering analysis revealed that cluster separation was driven more by task than by clinical group. Given we hypothesized significant between group EC differences between networks, this was surprising as we also anticipated within-cluster separation. However, this pattern is broadly consistent with the EC findings in suggesting that contextual demands shape network coordination more strongly than diagnostic status, at least as measured here. It is also consistent with the absence of VMN dominance in the observed EC analyses; if a single network (e.g., the VMN) was influencing dysfunction in another, we might expect EC patterns to show some degree of group-level separation. Instead, clustering by task rather than group suggests that network organization is dynamically shaped by cognitive and affective demands rather than fixed diagnostic differences. This finding underscores the importance of considering task context when characterising network organisation. This is consistent with emerging evidence that brain network dynamics differentiate dimensional symptom profiles more robustly than case-control or primary diagnostic groupings, with task-evoked reconfiguration representing a key axis of variation across health and disease (Cocuzza et al., 2025). However, alternative explanations for these findings include the clustering solution may be sensitive to noise in the EC estimates themselves, particularly given that no between-group EC differences survived correction in either task alongside the small sample size.

### 4.4 Implications

Although the present findings did not show between-group EC differences after correction for multiple comparisons in either task, the distributed uncorrected effects observed during CR may still have implications for better understanding large-scale coordination. Specifically, the findings suggest that changes in large-scale network coordination may be subtle and dependant on task context rather than reflecting a singular, consistently disrupted network. Such variability may also explain inconsistences in prior findings across the field, where differences in experimental design and task structure often yield heterogenous findings regarding the neural correlates of OUD.

### 4.5 Strengths and limitations

The methods and study design confer several strengths. Most previous neuroimaging studies in OUD have focused on regions and/or used FC which identified statistical dependencies between neural signals but does not infer directionality or causality (Zare Sadeghi et al., 2017). This limits our knowledge as to how different networks may influence one another. By evaluating group differences in lsNGC across different tasks, this study provides a more nuanced understanding as to how different brain networks communicate during different contexts and how disrupted connectivity across multiple functional systems may contribute to OUD related dysfunction. Also, using EC allowed an evaluation of the potential causal role of the VMN in broader network dysfunction in OUD. Despite the VMN not being identified as a dominant driver of EC differences, the present findings support consideration of more distributed models of network dysfunction involving interactions between salience, cognition, and sensorimotor systems rather than exclusively reward centred abnormalities. Finally, our clustering model enabled an exploratory, data-driven characterisation of EC patterns without relying on predefined group labels. That cluster separation was shaped more strongly by task than clinical status further emphasizes the importance of considering contextual factors in shaping large-scale network organization in OUD.

Limitations include the small sample size of our cohort which may have limited sensitivity to detect EC differences after correction for multiple comparisons. Additionally, although HC and OUD participants were well matched on some demographic and clinical factors, within- and between-group variability remained in factors such as smoking severity, education, socioeconomic status, and psychiatric diagnosis. Another limitation of the current study is that granger causality estimates were derived from BOLD fMRI signals without explicit modelling of the hemodynamic response function (HRF). Because regional variability in HRF timing and shape can induce spurious directionality, Granger causality applied to fMRI data may not fully reflect underlying neural interactions. While the use of a multivariate nonlinear framework (lsNGC) and a focus on network-level effects may reduce sensitivity to these confounds, the inferred directionality should be interpreted as reflecting statistical dependencies in BOLD signals rather than definitive neural causation. Lastly, the cross-sectional design of the study limits causal interpretation of the observed EC differences. As all OUD participants were receiving methadone maintenance therapy, it is not possible to determine whether the observed network level patterns reflect OUD pathology itself, treatment-related neural adaptations associated with methadone exposure, other clinical characteristics, or a combination of these factors. Consequently, the findings should not be generalised to untreated OUD populations.

Nonetheless, to date, EC studies remain underutilized compared to FC studies, especially in OUD, so the present findings offer valuable insight into large scale directed network organisation in OUD. We hope future work employs EC to evaluate whether the observed EC patterns are specific to OUD or generalisable across all SUDs. Future work could further increase the specificity of the findings by examining EC during task-relevant epochs, or by modeling EC across different task phases to isolate event-specific connectivity patterns. This could clarify whether distributed patterns of network organization vary according to the cognitive and affective demands and provide a better understanding of the contextual and clinical features of network reorganization in OUD.

### 4.6 Conclusion

In this study, we investigated whether disruptions in between network EC inn OUD would concentrate in the VMN, providing evidence for the urge-to-action framework proposed by Dunlop et al. (2017). However, we found no evidence that the VMN or any single network drove EC differences between HC and OUD participants in either task, or no between-group EC differences survived correction for multiple comparisons. Exploratory clustering further revealed that network organisation was shaped more strongly by task context rather than diagnostic status. Together, these null findings do not support a reward-centric, VMN-led model of hierarchical network dysfunction in OUD. Instead, our findings motivate a more cautious perspective that considers network disruption as distributed and dependent on cognitive context rather than reflecting a singular network abnormality.

## Supplementary Material

Supplementary Material

## Data Availability

The code to compute EC, make figures, and conduct statistical analysis described in this paper is available at: https://github.com/BriannaAus7/EffectiveConnectivityPublication
