# Supplementary material for: Investigating hierarchical control among functional networks disrupted by opioid use disorder using effective connectivity
Source: Imaging Neurosci (Camb). 2026 Sep 17;4:IMAG.a.1373. doi: 10.1162/IMAG.a.1373 (PMC13592366; doi:10.1162/IMAG.a.1373)
Supplement: Supplementary file 1 [file IMAG.a.1373_supp.pdf]

SUPPLEMENTARY MATERIALS

Investigating hierarchical control among functional networks disrupted by Opioid Use Disorder using effective connectivity

Austin *et al.*

1. Methods

1.1. Large scale nonlinear granger causality

EC was quantified using large-scale nonlinear granger causality (lsNGC), which infers directional and causal influence between timeseries (Wismüller et al., 2021). Granger causality relies on the premise that if the prediction quality of one time series improves when using past values of another time series, then the latter is considered to have a causal influence (Granger, 1969).

Unlike classical linear GC, lsNGC estimates conditional GC using a phase-space reconstructions of multivariate time series, whilst considering confounding variables and limited observations (Wismüller et al., 2021).

In the phase space reconstruction, *t* is time and *d* is the embedding dimension, determined by Cao's method (Cao, 1997). The embedding dimension is handled within the autoregressive order and forms a vector that encapsulates the historical values of X_a up to time, *t*. The autoregressive order determines how many past time points are used to predict the current value in a time series (Wismüller et al., 2021).

Phase space reconstruction occurs while splitting the input dataset into training, validation and test sets (Wismüller *et al.*, 2021). The split constructs the phase space by segmenting the data based on the autoregressive order. Phase space vectors are constructed by selecting autoregressive order consecutive times steps from the input data.

GC quantifies causal influence by comparing the prediction quality of estimation models: one using information from only the suspected influential timeseries (X_a) and another containing all states but X_a. If the model including X_a has a better prediction quality compared to the prediction quality of the model excluding X_a, then ***X_a is considered to Granger-cause X_b***.

LsNGC outputs an F-statistic and affinity matrix https://doi.org/10.1038/s41398-026-03845-6. The F-statistic quantifies X_a's directional influence on X_b by comparing the variance of precision errors from both models shown in equations (4) and (5). The F-statistic tests whether X_b's prediction error variance decreases when X_a is included in the model. If the variance is lower when X_a is included, then ***X_a Granger Causes X_b.***

The F-statistic is given by:

$$F_{X_a \to X_b} = \frac{\frac{RSS_R - RSS_U}{p_U - p_R}}{\frac{RSS_U}{n - p_U - 1}} \qquad (1)$$

Where RSS_R is the residual sum of squares for the restricted model, RSS_U is the residual sum of squares for the unrestricted model, pR is the number of parameters in the restricted model, pU is the number of parameters in the unrestricted model and n is the number of time-delayed vectors (n=(t=d-1).

For this study, *N*=214 brain regions, whilst *T* was timepoints. T=322 in the CR task and T= 292 in the MID task.

1.1.1. Nonlinear transformations using generalised radial basis functions (GRBF)

IsNGC uses GRBF neural networks to create nonlinear transformations, *f* and *g* (Wismüller *et al.*, 2021). K-means clustering determines the cluster centres (c_f and c_g) for the state spaces created.

Full IsNGC implementation and mathematical formulation are available in Wismüller *et al*. (2021)

1.2. Uniform Manifold Approximation and Projection

Uniform Manifold Approximation and Projection (UMAP) was used to reduce the dimensionality of the pairwise EC matrices for subsequent clustering analysis. UMAP is a nonlinear dimensionality reduction technique that preserves both global and local structure in high-dimensional data, making it well-suited for exploring patterns in brain connectivity networks. The pairwise connectivity values from flattened affinity matrices were scaled using MinMaxScaler prior to UMAP processing to ensure a normalized input range between 0 and 1.

The UMAP parameters were optimized through hyperparameter tuning. The optimised target was a combination of trustworthiness score and silhouette score. Trustworthiness was used to ensure that local neighbourhood structures were preserved in the low-dimensional embedding, while silhouette score was used to assess cluster separation quality. The selected UMAP parameters (n_neighbors=10, min_dist=0.1, n_components=10) provided a balance between preserving local relationships in the data structure and achieving meaningful cluster separation for consequent analysis.

UMAP was initialized with a fixed random seed (random_state=42) for reproducibility. The reduced embeddings were then used as inputs for subsequent clustering analysis described below.

1.3. Hierarchical Density-Based Spatial Clustering of Applications with Noise (HDBSCAN)

Following dimensionality reduction, HDBSCAN identified clusters within the UMAP-reduced embeddings. HDBSCAN is an unsupervised**,** density-based clustering algorithm that detects clusters of varying densities without a prior specification of the number of clusters (Campello, Moulavi, and Sander, 2013). In HDBSCAN, density is defined by the concentration of data points within a given neighbourhood, where clusters are formed in areas with a high density of points (Campello, Moulavi, and Sander, 2013). HDBSCAN identifies clusters based on how closely data points are

packed together, which allows for structures of varying densities to be identified (Campello, Moulavi, and Sander, 2013). HDBSCAN uses three density-related categories to classify data points (Campello, Moulavi, and Sander, 2013). Firstly, core points which are data points that are surrounded by a sufficient number of neighbours within a specified radius. These points are essential for defining dense regions. Secondly, border points which are data points that are within a cluster but do not meet the density threshold to be core points. Lastly, noise points, which are data points that do not belong to any dense region and do not fit into any well-defined clusters.

An advantage of HDBSCAN is its ability to classify noise points which may represent measurement artifact or individuals whose connectivity patterns do not conform to dominant group structures. As the clustering is independent of the participants' clinical status, HDBSCAN identified clusters based solely on the structure of the data rather than predefined categories. This data-driven approach allowed for the discovery of natural connectivity patterns that may reflect underlying neurobiological heterogeneity.

The HDBSCAN parameters were a minimum cluster size of 5 and a minimum number of samples per cluster of 10, using Euclidean distance as the similarity metric. These parameters were chosen through a hyperparameter search to optimise cluster separability while maintaining the ability to detect subtle structure in the data.

2. Results

Table 1: The 10 most important features for each cluster, determined using the Kruskal-Wallis test to identify significant differences in feature distributions across clusters. Each feature represents a connection (edge) between two brain regions, defined by their hemisphere (LH = left hemisphere, RH = right hemisphere), functional network, and anatomical region.

Feature Importance for each cluster		
**Cluster 0**	**Cluster 1**	**Noise**
RH Salience/Ventral Attention Network Frontal Operculum – RH Somatomotor Network	RH Dorsal Attentional Network Postcentral Gyrus - LH Salience/Ventral Attention Network Frontal Operculum	RH Default Mode Network Dorsal Prefrontal Cortex – RH Visual Central Network Extrastriate Cortex
RH Salience/Ventral Attention Network Frontal Operculum – RH Salience/Ventral	LH Salience/Ventral Attention Network Frontal Medial Cortex – RH Default Mode Network Retrosplenial Cortex	LH Somatomotor Network – RH Limbic Network Temporal Pole

Attention Network Frontal Medial Cortex		
LH Somatomotor Network – LH Dorsal Attentional Network Frontal Eye Fields	LH Somatomotor Network, Central Region - LH Salience/Ven tral Attention Network Frontal Medial Cortex	RH Visual Periphery Netw ork, Striate Cortex (Calcarine C ortex) – RH Somatomotor Network
RH Salience/Ventral Attention Insula – RH Visual Peripheral Extra-Striate Cortex, Superior Part	RH Salience/Ventral Attention Network Frontal Medial Cortex – RH Default Mode Network Retrosplen ial Cortex	RH Control Network Precu neus – RH Visual Central Network Extrastriate Cortex
RH Somatomotor Network, Secondary Somatosensory Cortex – LH Salience/Ventral Attention Network Medial Parietal Cortex	RH Somatomotor Network, Secondary Somatosensory Corte x – RH Control Network, Dorsal Prefrontal Cortex	RH Default Mode Network Inferior Parietal Lobule – L H Visual Central Network Extrastriat e Cortex
LH Somatomotor Netw ork Auditory Cortex – RH Dorsal Attentional Network Postcentral G yrus	RH Salience/Ventral Attention Network Frontal Operculum - RH Salience/Ventral Attention Networ k Frontal Medial Cortex	LH Control Network Intrap arietal Sulcus – LH Visual Central Network Extrastriate Corte x
LH Salience/Ventral Attention Network Insula – LH Dorsal Attention Network Frontal Eye Fields	RH Somatomotor Network – LH Salience/Ventral Attention Networ k Frontal Operculum	LH Salience/Ventral Attenti on Network Insula – RH Visua l Central Network Extrastriat e Cortex
LH Salience/Ventral Att ention Network Frontal Operc ulum - LH Salience/Ventral Attent ion Network Medial Parietal Cortex	RH Somatomotor Network - RH Control Network, Dorsal Prefronta l Cortex	RH Visual Periphery Netw ork, Striate Cortex (Calcarine C ortex)- RH Control Networ k Temporal Cortex
LH Salience/Ventral Att ention Network Insula - LH Salience/Ventral	LH Salience/Ventral Attention Network Frontal Medial Cortex – RH Control Network Precuneus	RH Visual Periphery Netw ork, Striate Cortex (Calcarine C ortex) -

Attention Network Medi al Parietal Cortex		RH Somatomotor Network
RH Salience/Ventral At tention Network Insula – RH S omatomotor Network	LH Somatomotor Network Central Region – RH Default Mode Netw ork Retrosplenial Cortex	LH Visual Periphery Netwo rk, Striate Cortex (Calcarine C ortex) – LH Default Mode Network Temporal Cortex

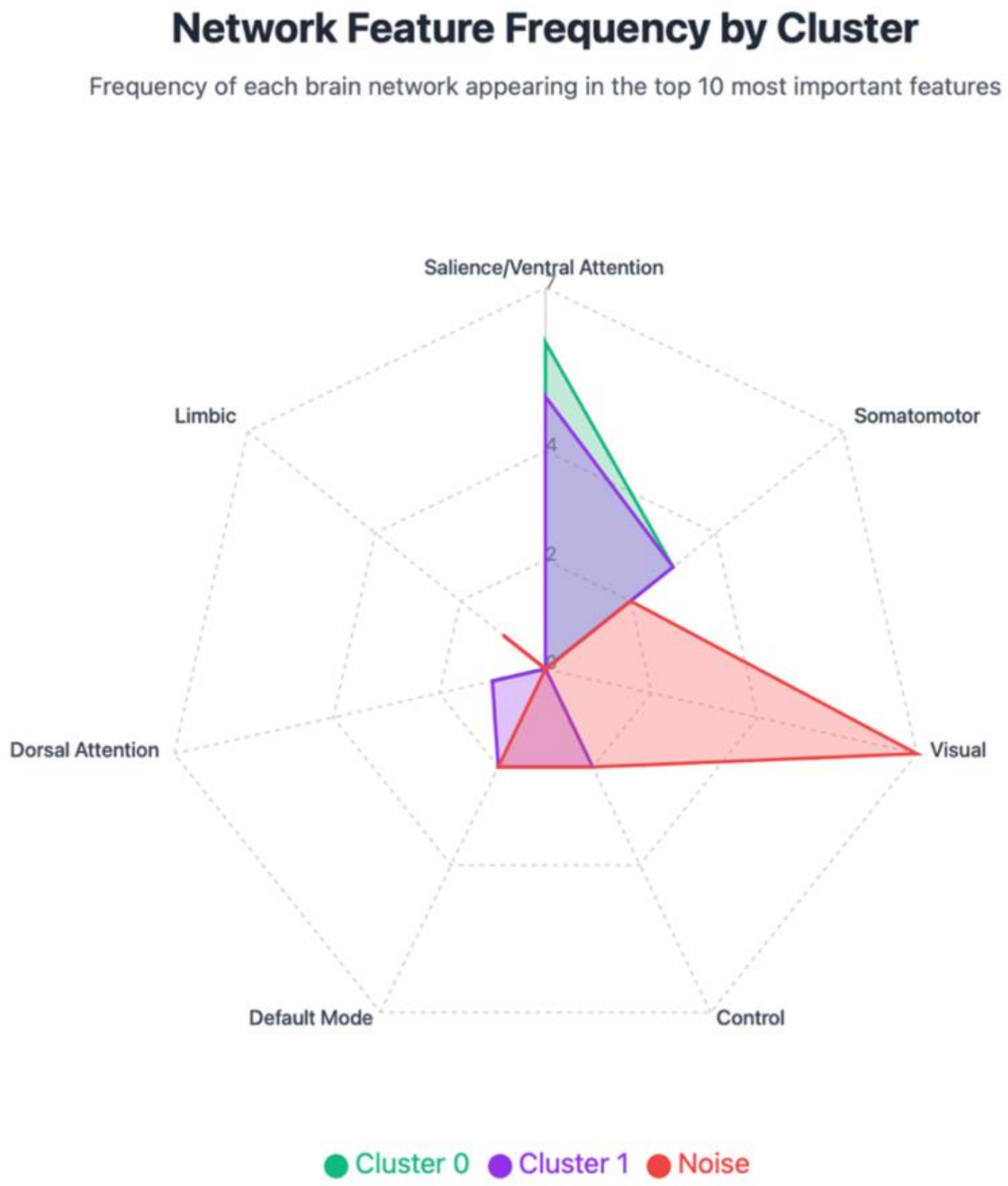

Figure 1: Radar plot showing the frequency of functional brain networks across the clusters. The plot is showing the occurrence of each brain network among the top 10 most important features identified by the Kruskal-Wallis test. Each axis represents the network, and the radial distance reflects the number of times the network appears in the top 10 features.

Table 2: The between-network EC pairs showing the corrected and uncorrected group differences for the Cue task.

Network Pair	Uncorrected p-value	Corrected p-value	F-Statistic	Partial Eta2	Hedges G	HC Mean	HC SD	OUD Mean	OUD SD
**Visual-->Visual**	0.096336183	0.28063202	2.884588425	0.060240435	-0.477668362	0.123238114	0.06686	0.096296035	0.04307267
**Visual-->Somatomotor**	0.210074356	0.301663574	1.6167785	0.03468233	-0.357610129	0.124932259	0.079705333	0.099568072	0.059666449
**Visual-->Dorsal Attention**	0.345123409	0.367631457	0.910333291	0.019828505	-0.268339596	0.112236189	0.064920051	0.095347088	0.059100501
**Visual-->Salience Ventral Attention**	0.114543682	0.28063202	2.589861346	0.054420443	-0.452608604	0.126127347	0.074632043	0.09655703	0.053512199
**Visual-->Control**	0.191011554	0.301663574	1.762477351	0.037689991	-0.373375933	0.099458232	0.050181705	0.081048998	0.046937494
**Visual-->Default Mode**	0.256883324	0.307006899	1.318756613	0.02847133	-0.322973488	0.094057338	0.053719915	0.077726059	0.045931025
**Visual-->Limbic**	0.206781085	0.301663574	1.640778274	0.03517905	-0.360254569	0.077001337	0.05719653	0.059744135	0.03601387
**Somatomotor-->Visual**	0.390362898	0.398495458	0.7522612	0.016442055	-0.243932058	0.129967833	0.092769771	0.109464729	0.0726347
**Somatomotor-->Somatomotor**	0.550545891	0.550545891	0.361764924	0.007975107	-0.169160039	0.387567599	0.219663748	0.352857918	0.184654335
**Somatomotor-->Dorsal Attention**	0.309625601	0.344810328	1.055981673	0.02292822	-0.289009913	0.232824862	0.147840174	0.191427943	0.134409057
**Somatomotor-->Salience Ventral Attention**	0.099496137	0.28063202	2.829134914	0.059150869	-0.473054718	0.301468639	0.186955409	0.224345181	0.132654281
**Somatomotor-->Control**	0.213927075	0.301663574	1.589276404	0.034112494	-0.354555533	0.17163973	0.115898292	0.13415981	0.092208647
**Somatomotor-->Default Mode**	0.252882747	0.307006899	1.341483062	0.02894778	-0.325744541	0.163327972	0.113857352	0.130344038	0.085101749
**Somatomotor-->Limbic**	0.082452425	0.28063202	3.155079089	0.065519134	-0.49956232	0.123120755	0.093308374	0.085694253	0.050557292
**Dorsal Attention-->Visual**	0.382712938	0.398495458	0.777103405	0.01697581	-0.247927074	0.10314806	0.078819374	0.085760306	0.058990283
**Dorsal Attention-->Somatomotor**	0.092496738	0.28063202	2.95478709	0.061616103	-0.483445637	0.226168565	0.13831238	0.164449743	0.113156428
**Dorsal Attention-->Dorsal Attention**	0.249480864	0.307006899	1.361168104	0.029360091	-0.328125839	0.226320273	0.122267909	0.185786988	0.120747448

**Dorsal Attention-->Salience Ventral Attention**	0.043490848	0.28063202	4.316036354	0.087517908	-0.584288174	0.202035469	0.133503962	0.13371042	0.09587354
**Dorsal Attention-->Control**	0.136355707	0.29049694	2.300148723	0.048628784	-0.426542694	0.155692544	0.096660178	0.118296547	0.075879802
**Dorsal Attention-->Default Mode**	0.269399706	0.314299657	1.250455645	0.027036612	-0.314498584	0.1179474	0.090820793	0.091223119	0.076624383
**Dorsal Attention-->Limbic**	0.178867058	0.301663574	1.864731572	0.039789657	-0.38405437	0.099026264	0.087296603	0.070526282	0.057567743
**Salience Ventral Attention-->Visual**	0.108377181	0.28063202	2.68334361	0.056274233	-0.460704754	0.123547742	0.080264054	0.090772282	0.059474931
**Salience Ventral Attention-->Somatomotor**	0.02235998	0.28063202	5.596916521	0.110617739	-0.665363486	0.279869063	0.167080165	0.18727759	0.103328218
**Salience Ventral Attention-->Dorsal Attention**	0.105069312	0.28063202	2.736011342	0.057315458	-0.465204059	0.2015938	0.128255043	0.146545028	0.104828372
**Salience Ventral Attention-->Salience Ventral Attention**	0.046127998	0.28063202	4.206131238	0.08547982	-0.576800939	0.316709952	0.19484756	0.22182362	0.125834402
**Salience Ventral Attention-->Control**	0.111641517	0.28063202	2.633126124	0.055279305	-0.456373459	0.210691786	0.130218732	0.157978059	0.096678056
**Salience Ventral Attention-->Default Mode**	0.126340564	0.29049694	2.426030982	0.051153996	-0.438059114	0.175749651	0.109591836	0.132436015	0.084926942
**Salience Ventral Attention-->Limbic**	0.155029146	0.301663574	2.091717806	0.044417955	-0.406757993	0.128959649	0.098342113	0.095668878	0.060662083
**Control-->Visual**	0.048811489	0.28063202	4.101121467	0.083523988	-0.569555258	0.090921295	0.062620953	0.061653535	0.036815707
**Control-->Somatomotor**	0.026174599	0.28063202	5.287428496	0.105144141	-0.646705869	0.157902139	0.102912471	0.102419651	0.063834396
**Control-->Dorsal Attention**	0.085540895	0.28063202	3.090707935	0.064268298	-0.494439926	0.155768878	0.095457185	0.113728898	0.07163362
**Control-->Salience Ventral Attention**	0.029923101	0.28063202	5.02742007	0.100493291	-0.630604603	0.198544084	0.126501728	0.132122375	0.078155682
**Control-->Control**	0.086380978	0.28063202	3.073647172	0.063936218	-0.493073379	0.19981692	0.111748748	0.152261192	0.077038679
**Control-->Default Mode**	0.150361258	0.301663574	2.14097239	0.045416382	-0.411519181	0.163019408	0.100441618	0.126240821	0.07517497
**Control-->Limbic**	0.134815347	0.29049694	2.318803802	0.049003855	-0.428268913	0.104722734	0.080489714	0.076332421	0.047927037
**Default Mode-->Visual**	0.170655141	0.301663574	1.938674707	0.041302289	-0.391594895	0.095169563	0.073811823	0.070881111	0.046970885

**Default Mode-->Somatomotor**	0.095297151	0.28063202	2.903270929	0.060606945	-0.479212714	0.167088966	0.111663997	0.120945841	0.076787245
**Default Mode-->Dorsal Attention**	0.285188148	0.324981843	1.16988043	0.025338606	-0.304197252	0.126332986	0.097408075	0.098690347	0.081644887
**Default Mode-->Salience Ventral Attention**	0.068296309	0.28063202	3.489047101	0.071955365	-0.52533697	0.185302212	0.118366775	0.130885346	0.084795563
**Default Mode-->Control**	0.195713109	0.301663574	1.724961932	0.036917353	-0.369380791	0.17678321	0.104493523	0.141073014	0.085951398
**Default Mode-->Default Mode**	0.330329132	0.359691722	0.96844872	0.021067683	-0.276772454	0.19927687	0.110354939	0.168646522	0.10744942
**Default Mode-->Limbic**	0.245144078	0.307006899	1.386757795	0.029895553	-0.331195828	0.131225174	0.083313534	0.104371065	0.076443742
**Limbic-->Visual**	0.234105806	0.307006899	1.454527149	0.031310773	-0.339191894	0.079666746	0.063899871	0.060951278	0.044111587
**Limbic-->Somatomotor**	0.028034448	0.28063202	5.153730964	0.102758675	-0.638477241	0.134731156	0.09928609	0.084902966	0.04914025
**Limbic-->Dorsal Attention**	0.217214817	0.301663574	1.566281106	0.033635521	-0.351981148	0.109014254	0.093708375	0.080586945	0.064343556
**Limbic-->Salience Ventral Attention**	0.091495826	0.28063202	2.973626134	0.061984602	-0.484984358	0.144512608	0.107230644	0.10135131	0.065540614
**Limbic-->Control**	0.18164851	0.301663574	1.840595571	0.03929488	-0.381560787	0.116433385	0.08527822	0.088217742	0.059572971
**Limbic-->Default Mode**	0.221630381	0.301663574	1.536059343	0.033007938	-0.348568828	0.139186587	0.08668022	0.109510492	0.081021971
**Limbic-->Limbic**	0.178914793	0.301663574	1.864313595	0.039781093	-0.384011325	0.156172132	0.119557662	0.113319065	0.100329875

Table 3: The between-network EC pairs showing the corrected and uncorrected group differences for the MID task

Network Pair	Uncorrected p-value	Corrected p-value	F-Statistic	Partial Eta^2	Hedges G	HC Mean	HC SD	OUD Mean	OUD SD
**Visual --> Visual**	0.8952239	0.962638885	0.017541302	0.000389655	-0.037249072	0.101442062	0.075723276	0.098894781	0.058819734

**Visual --> Somatomotor**	0.844306615	0.962638885	0.039015945	0.00086627	-0.055552756	0.089904634	0.062890719	0.086935709	0.04143088
**Visual-->Dorsal Attention**	0.825994911	0.962638885	0.048896556	0.001085411	-0.062190448	0.102415469	0.081512772	0.098028322	0.056629083
**Visual-->Salience Ventral Attention**	0.696035163	0.962638885	0.154599066	0.003423772	0.110582902	0.081105527	0.055924244	0.087114228	0.051139918
**Visual-->Control**	0.94403215	0.962638885	0.004983787	0.000110739	-0.019854743	0.080109271	0.054327325	0.079096124	0.046232219
**Visual-->Default Mode Network**	0.791058323	0.962638885	0.071031473	0.00157599	0.074956614	0.07833375	0.06298301	0.082864535	0.056141662
**Visual-->Limbic**	0.16200714	0.962638885	2.021233338	0.042985545	0.399846018	0.063839257	0.036805507	0.081692257	0.049279098
**Somatomotor-->Visual**	0.484733169	0.962638885	0.49636259	0.01090994	-0.198145435	0.086195377	0.065482453	0.075307083	0.041492535
**Somatomotor-->Somatomotor**	0.089557979	0.962638885	3.010766107	0.062710228	-0.488003636	0.172746909	0.086144642	0.136648482	0.058526293
**Somatomotor-->Dorsal Attention**	0.349433475	0.962638885	0.89404382	0.019480607	-0.265927929	0.134844548	0.091132325	0.114876587	0.054343838
**Somatomotor-->Salience Ventral Attention**	0.256464759	0.962638885	1.321113379	0.02852076	-0.323261954	0.143663242	0.093198009	0.117926348	0.062369541
**Somatomotor-->Control**	0.900479238	0.962638885	0.015816491	0.000351354	-0.035370372	0.093819091	0.064930042	0.091667865	0.054920932
**Somatomotor-->Default Mode Network**	0.842996004	0.962638885	0.039684561	0.000881102	-0.056026738	0.097750207	0.078139798	0.093940952	0.055106679
**Somatomotor-->Limbic**	0.355067993	0.962638885	0.873164841	0.019034327	0.262804422	0.081785795	0.059817013	0.096796917	0.05275592
**Dorsal Attention-->Visual**	0.761976623	0.962638885	0.092860457	0.002059316	-0.085703776	0.083746202	0.064566618	0.078776638	0.049467441
**Dorsal Attention-->Somatomotor**	0.47603195	0.962638885	0.516551587	0.011348654	-0.202134944	0.124765576	0.070104895	0.112392453	0.049916139
**Dorsal Attention-->Dorsal Attention**	0.493130479	0.962638885	0.477446359	0.010498531	-0.194333128	0.149892595	0.100012291	0.133422688	0.06532836
**Dorsal Attention-->Salience Ventral Attention**	0.594348401	0.962638885	0.287686915	0.006352431	-0.150849753	0.112436979	0.072379401	0.102822017	0.052728413

**Dorsal Attention-->Control**	0.576089407	0.962638885	0.317202549	0.006999606	-0.158399163	0.107910262	0.068927354	0.098162142	0.052039844
**Dorsal Attention-->Default Mode Network**	0.845380479	0.962638885	0.038472508	0.000854214	-0.055164514	0.082186269	0.07120027	0.078843283	0.047128282
**Dorsal Attention-->Limbic**	0.162740662	0.962638885	2.014031465	0.042838944	0.399133035	0.063406382	0.045418798	0.085088782	0.059534256
**Salience Ventral Attention-->Visual**	0.453183944	0.962638885	0.572567815	0.01256387	-0.212812932	0.096550083	0.065141101	0.083999949	0.050903139
**Salience Ventral Attention-->Somatomotor**	0.116930358	0.962638885	2.555205917	0.053731361	-0.449570185	0.16925047	0.092987018	0.13278011	0.066055144
**Salience Ventral Attention-->Dorsal Attention**	0.28265281	0.962638885	1.182425907	0.025603374	-0.305823967	0.148402903	0.100604359	0.122571545	0.063844381
**Salience Ventral Attention-->Salience Ventral Attention**	0.285718301	0.962638885	1.167275391	0.02528361	-0.303858377	0.181245718	0.108214106	0.151555984	0.084019379
**Salience Ventral Attention-->Control**	0.435357125	0.962638885	0.61950527	0.013579833	-0.221364029	0.127171111	0.088174477	0.110417824	0.059830702
**Salience Ventral Attention-->Default Mode Network**	0.722481552	0.962638885	0.127717089	0.002830125	-0.100509949	0.114149258	0.089578203	0.106360663	0.062157715
**Salience Ventral Attention-->Limbic**	0.539134105	0.962638885	0.382977975	0.008438802	0.174048966	0.08604121	0.067978377	0.096916991	0.055085176
**Control-->Visual**	0.73539833	0.962638885	0.115636521	0.002563114	-0.095638346	0.075400443	0.049359748	0.070847612	0.044452897
**Control-->Somatomotor**	0.844428876	0.962638885	0.038953874	0.000864893	-0.055508549	0.095377149	0.057948576	0.09226613	0.052493524
**Control-->Dorsal Attention**	0.341638414	0.962638885	0.923711463	0.020114042	-0.270304151	0.125185624	0.079268867	0.10662966	0.055175494
**Control-->Salience Ventral Attention**	0.645261753	0.962638885	0.214804076	0.004750747	-0.130348431	0.109561943	0.071632092	0.101124587	0.055723717

**Control-->Control**	0.379717583	0.962638885	0.787027122	0.017188867	-0.249505083	0.142069681	0.085358088	0.123542029	0.060168102
**Control-->Default Mode Network**	0.901815879	0.962638885	0.015392269	0.000341933	-0.034892804	0.108798853	0.072096398	0.106498674	0.057697916
**Control-->Limbic**	0.314828542	0.962638885	1.033259794	0.02244594	0.285883647	0.075452017	0.04723695	0.09010774	0.053022807
**Default Mode Network-->Visual**	0.868348257	0.962638885	0.027790874	0.000617194	0.046885187	0.077357033	0.057281358	0.080027877	0.054875105
**Default Mode Network-->Somatomotor**	0.957598773	0.962638885	0.002858411	6.35000000000000E-05	-0.015036505	0.100265222	0.074790784	0.099279494	0.053810876
**Default Mode Network-->Dorsal Attention**	0.69832863	0.962638885	0.152150817	0.003369736	-0.109703806	0.097135185	0.079459993	0.089607327	0.054869571
**Default Mode Network-->Salience Ventral Attention**	0.962638885	0.962638885	0.002218773	4.93036411476889E-05	0.013247716	0.103174049	0.080847064	0.104130142	0.061010136
**Default Mode Network-->Control**	0.958103369	0.962638885	0.002790719	6.20121274436722E-05	0.014857392	0.112479075	0.076346219	0.113528209	0.062756313
**Default Mode Network-->Default Mode Network**	0.895560246	0.962638885	0.017428192	0.000387143	0.037128784	0.142146591	0.088319223	0.145387012	0.083557389
**Default Mode Network-->Limbic**	0.303363772	0.962638885	1.084010528	0.023522487	0.292820379	0.095721662	0.062944219	0.114116801	0.060719541
**Limbic-->Visual**	0.272692984	0.962638885	1.23315495	0.026672524	0.312315382	0.065476312	0.036557849	0.07904935	0.047478645
**Limbic-->Somatomotor**	0.465886518	0.962638885	0.540874582	0.011876684	0.206839193	0.089613062	0.061727389	0.101419162	0.050711582
**Limbic-->Dorsal Attention**	0.461268072	0.962638885	0.552235046	0.012123116	0.209000118	0.078621628	0.059809404	0.091742889	0.063360811
**Limbic-->Salience Ventral Attention**	0.592670209	0.962638885	0.290321673	0.006410237	0.151538952	0.088716754	0.071797379	0.098296346	0.05228084
**Limbic-->Control**	0.421993303	0.962638885	0.65669746	0.014383376	0.227912013	0.081991028	0.054865861	0.094488608	0.053071082
**Limbic-->Default Mode Network**	0.425347826	0.962638885	0.647193378	0.014178164	0.226256769	0.101071558	0.06469519	0.115780903	0.063237996

**Limbic-->Limbic**	0.181753318	0.962638885	1.839694717	0.039276403	0.3814674	0.112094702	0.072222732	0.144021103	0.090183279
